# Assessing the real-world safety of fenofibric acid for hyperlipidemia: results from WHO-VigiAccess and FAERS databases

**DOI:** 10.3389/fmed.2025.1702197

**Published:** 2025-11-07

**Authors:** Yaxing Li, Yi Wang, Jidang Zhang, Ruonan Zhang, Zhiwen Yao, Xuepin Chen, Xingli Xu

**Affiliations:** 1 State Key Laboratory for Innovation and Transformation of Luobing Theory, First Clinical College, Shandong University, Jinan, China; 2 Key Laboratory of Cardiovascular Remodeling and Function Research of MOE, NHC, CAMS and Shandong Province, Department of Cardiology, Qilu Hospital of Shandong University, Jinan, China; 3 Nanjing University of Chinese Medicine, Nanjing, China; 4 Second Clinical Medical College, Shandong University of Traditional Chinese Medicine, Jinan, China; 5 Medical School, Shandong University of Traditional Chinese Medicine, Jinan, China; 6 Department of Cardiology, Qingdao Hospital, University of Health and Rehabilitation Sciences, Qingdao, China; 7 Department of Cardiology, Sichuan Provincial People’s Hospital, University of Electronic Science and Technology of China, Chengdu, China

**Keywords:** fenofibric acid, hyperlipidemia, WHO-VigiAccess, FAERS, disproportionality analysis, pharmacovigilance, adverse drug reaction

## Abstract

**Background:**

Fenofibric acid is a small-molecule fibrate that functions as an agonist of peroxisome proliferator-activated receptor alpha (PPARα) and serves as an inhibitor of liver fatty acid-binding protein. It is primarily prescribed for the management of hyperlipidemia, including conditions such as hypercholesterolemia and hypertriglyceridemia. As a lipid-lowering agent, a comprehensive understanding of the real-world safety profile of fenofibric acid is essential to ensure its safe and effective use in clinical practice.

**Methods:**

This study utilizes four disproportionality analysis methods to investigate adverse event (AE) reports related to fenofibric acid in the WHO VigiAccess and FDA Adverse Event Reporting System (FAERS) databases, thereby providing robust scientific evidence for evaluating the real-world safety of fenofibric acid. Additionally, the study applies the Weibull distribution to estimate the timing of adverse event occurrences. The study investigates the relationship between adverse event reports and gender via gender-stratified analysis.

**Results:**

This study retrieved 323 adverse event reports from WHO VigiAccess and 1,970 reports from FAERS. Drug-related signals were detected in 23 and 26 System Organ Class levels in the WHO VigiAccess and FAERS datasets, respectively. The study results confirmed known adverse reactions of fenofibric acid, including renal impairment, hepatobiliary toxicity, pancreatitis, and allergic reactions. Additionally, several potential adverse effects were identified, including gout, hypoglycemia, prothrombin time prolonged, photosensitivity reactions, rash, blood creatine and creatinine increased, blood creatine phosphokinase increased, myalgia, muscle fatigue, pain in extremity, joint pain and headache. The findings further underscore the importance of monitoring adverse events during the first 3 months of fenofibric acid use. The findings also highlight that closer attention to adverse events among female patients may have important clinical implications.

**Conclusion:**

In addition to the known adverse reactions, this study has identified numerous potential adverse drug reactions associated with fenofibric acid. Although these findings require further validation through subsequent clinical trials, they provide valuable safety information for clinicians to consider when evaluating adverse effects in patients treated with fenofibric acid.

## 1 Introduction

Hyperlipidemia is a major public health concern, with its incidence and prevalence steadily increasing worldwide (1). It is primarily characterized by elevated plasma triglycerides, total cholesterol, and low-density lipoprotein (LDL) levels, along with decreased high-density lipoprotein (HDL) levels. Hyperlipidemia can be classified into two types: primary hyperlipidemia and acquired hyperlipidemia. More than 60% of lipid metabolism disorders result from a combination of genetic predisposition and environmental factors, known as primary hyperlipidemia (2). In contrast, acquired hyperlipidemia (also referred to as secondary dyslipidemia) is caused by underlying diseases that alter plasma lipid and lipoprotein metabolism (3). Hyperlipidemia poses a serious threat to human health. Previous studies have shown that it is a common risk factor for vascular diseases and a key determinant in the development of coronary atherosclerotic heart disease and stroke (4–8). Additionally, reports of primary hyperlipidemia cases associated with spastic-ataxic syndrome suggest a potential neurological impact of hyperlipidemia (9).

Screening and treatment for hyperlipidemia are essential (10). It is strongly recommended that men over 35 years old and women over 45 years old with an increased risk of cardiovascular events undergo lipid disorder screening (Grade A recommendation) (8). The primary goal of hyperlipidemia treatment is to reduce plasma lipoprotein concentrations, thereby decreasing the amount of lipids entering the arterial walls to prevent the onset and progression of atherosclerosis. The secondary objective is to prevent adverse complications (11). Previous studies have confirmed that lowering serum cholesterol provides clinical benefits in both the primary and secondary prevention of coronary artery disease (12). When treating hyperlipidemia, the first approach should focus on lifestyle modifications to correct lipid abnormalities. This includes reducing the intake of saturated fats and cholesterol, increasing physical activity, and other behavioral changes. If lipid levels remain abnormal after 3 months, pharmacological intervention should be considered (8, 13). Statins are the primary class of drugs used to treat hyperlipidemia (14). They work by inhibiting 3-hydroxy-3-methylglutaryl coenzyme A (HMG-CoA) reductase, thereby reducing cholesterol synthesis and increasing LDL receptor expression (8).

Fenofibric acid is another commonly used drug for treating hyperlipidemia. It primarily corrects lipid abnormalities by targeting the activation of PPARα and inhibiting liver fatty acid-binding protein (L-FABP). PPARα is a ligand-activated transcription factor expressed in the liver. Upon activation, it effectively induces the expression of genes involved in lipid metabolism, thereby helping to correct lipid imbalances (15). L-FABP has been shown to participate in fatty acid solubilization within the cytoplasm, bind lipophilic compounds, and exhibit specificity in interacting with fibrate drugs (16). Among them, FABP1 plays a crucial role in fatty acid uptake and intracellular transport, making it essential for regulating lipid metabolism and intracellular signaling pathways (17). Inhibiting L-FABP may help reduce the accumulation of total cholesterol and triglycerides, thereby improving lipid disorders. However, the role of L-FABP inhibition in correcting dyslipidemia remains controversial and requires further research for validation. As a small-molecule fibrate drug, fenofibric acid primarily exerts its lipid-lowering effects by activating PPARα. This leads to reductions in LDL, total cholesterol, triglycerides, and apolipoprotein B, while increasing HDL levels. It is clinically used in the treatment of severe hypertriglyceridemia, primary hypercholesterolemia, and mixed dyslipidemia (18).

Therefore, fenofibric acid is primarily used in clinical practice for the treatment of hyperlipidemia and the reduction of vascular disease risk. However, there is still a lack of comprehensive evaluations regarding the long-term safety of fenofibric acid in real-world clinical settings. Previous studies have utilized the FAERS and even combined multiple databases, such as WHO-VigiAccess and FAERS, to analyze the relationship between drugs and adverse reactions. For example, research has explored the association between sugammadex and airway spasm-related adverse events (19), as well as the link between bevacizumab and gastrointestinal perforations (20). By integrating two databases, studies have explored the relationship between gepant-class drugs for migraine treatment, such as rimegepant, atogepant, and ubrogepaaant, and adverse hepatic events (21). Additionally, research has investigated the association between antifibrotic drugs like pirfenidone and nintedanib and adverse reactions such as hemiplegia, headache, weakness, and abdominal bloating (22). The Uppsala Monitoring Centre (UMC), which maintains WHO-VigiAccess, takes great care to ensure that the data displayed in VigiAccess accurately reflects the publicly accessible information collected by national regulatory authorities and transmitted to UMC. FAERS supports the FDA’s post-marketing safety surveillance of all approved drugs and therapeutic biologics, incorporating adverse event reports from healthcare professionals, consumers, and other sources. Both databases encompass a wide range of drug users. As a result, WHO-VigiAccess and FAERS serve as valuable resources for assessing drug safety. In this study, we leverage WHO-VigiAccess and FAERS data in which fenofibric acid is the primary suspected drug and apply disproportionality analysis to evaluate its safety in real-world settings. The findings aim to provide evidence-based guidance for the safe clinical use of fenofibric acid.

## 2 Materials and methods

### 2.1 Data sources, management, and study design

The data used in this study were obtained from the publicly accessible WHO-VigiAccess and FAERS database. The data from WHO-VigiAccess spans from 2010 to 2024, while the FAERS data covers 83 quarters, ranging from the first quarter of 2004 to the third quarter of 2024. All adverse event reports included in this study were identified with fenofibric acid as the primary suspect drug.

A search of the WHO VigiAccess database was performed to identify adverse events associated with fenofibric acid via its generic name. The retrieved reports were analyzed according to patient age group, gender, reporting year, and geographic region. Adverse events were classified according to the Medical Dictionary for Regulatory Activities (MedDRA), with a focus on the Preferred Term (PT) level within relevant System Organ Class (SOC) categories. Serious outcomes were categorized as death, hospitalization, or major events, which included life-threatening events, disability, and congenital anomalies.

The FAERS data management process involved removing duplicate reports and standardizing adverse event terminology to ensure accuracy and reliability. The FAERS data files comprise seven databases, including demographic and administrative information (DEMO), adverse drug reaction information (REAC), patient outcome information (OUTC), drug information (DRUG), drug therapy start and end dates (THER), information on report sources (RPSR), and indications for use/diagnosis (INDI). Duplicate reports were eliminated following the FDA’s recommended methodology: the PRIMARYID, CASEID, and FDA_DT fields from the DEMO table were used. The reports were then organized by CASEID, FDA_DT, and PRIMARYID. For records sharing the same CASEID, only the report with the most recent FDA_DT was retained. If both CASEID and FDA_DT were identical, the report with the largest PRIMARYID was retained. During the study period, a total of 21,964,449 reports related to fenofibric acid were extracted from the FAERS database. After duplicate exclusion, 18,278,243 case reports identified fenofibric acid as the primary suspect drug, and 1,970 adverse events were associated with fenofibric acid. Furthermore, to enhance the reliability of subsequent statistical analyses and results, we described and validated adverse event reports from both databases based on the standardized SOC and PT in the MedDRA.

### 2.2 Statistical analysis

A retrospective quantitative analysis was conducted to examine all data from WHO-VigiAccess and FAERS and derive the results. The descriptive analysis section characterizes adverse event reports in which fenofibric acid is identified as the primary suspected drug. The other sections utilize four disproportionality analysis methods to detect signals of adverse reactions associated with fenofibric acid. The four disproportionality analysis methods are Reporting Odds Ratio (ROR) (23), Bayesian Confidence Propagation Neural Network (BCPNN) (24), Proportional Reporting Ratio (PRR) (25), Multi-item Gamma Poisson Shrinker (MGPS) (23).

We applied four disproportionality analysis methods in parallel (i.e., not emphasizing one over the others) to screen for potential adverse events associated with fenofibric acid. Additionally, to avoid false-positive adverse event signals, positive signals in the statistical analysis must meet the corresponding thresholds of the four disproportionality analysis methods. These values are calculated based on the two-by-two contingency table used in disproportionality analysis. Descriptions of the formulas and threshold values for these methods can be found in Supplementary Table 2, while the detailed two-by-two contingency table is provided in Supplementary Table 1. Additionally, we utilized the Weibull distribution to model changes in adverse event incidence over time. In the Weibull distribution, the time-to-onset of adverse events related to fenofibric acid was defined as the time difference between the adverse event occurrence date (as reported in the DEMO file) and the drug initiation date (as recorded in the THER file). All statistical analyses were performed using SAS version 9.4.

## 3 Results

### 3.1 Descriptive analysis

This investigation encompassed 323 adverse event reports from WHO-VigiAccess and 1970 reports of adverse events (4,021 total adverse events) from FAERS in which fenofibric acid was the suspected primary agent. In terms of gender distribution, WHO-VigiAccess reported 155 cases (47.99%) in females and 140 cases (43.34%) in males, with a male-to-female ratio of 0.903:1. In FAERS, there were 748 cases (37.97%) in females and 765 cases (38.83%) in males, resulting in a nearly equal male-to-female ratio of 1.023:1. These findings suggest that females slightly outnumber males in WHO-VigiAccess, whereas FAERS exhibits a more balanced gender distribution. This discrepancy may be due to the smaller dataset of WHO-VigiAccess or could indicate potential gender-related differences in adverse drug reactions. Regarding age distribution, the primary age groups in WHO-VigiAccess were 45–64 years (133 cases, 41.18%) and 65–74 years (64 cases, 19.81%). In FAERS, the main age groups were 45–64 years (314 cases, 15.94%) and ≥ 65 years (208 cases, 10.56%). The relatively lower proportion of these age groups in FAERS is primarily due to a large number of reports with unknown age, accounting for 1,385 cases (70.30%) of the total dataset.

The annual distribution of reports in WHO-VigiAccess shows that the highest proportion occurred in 2020, with 69 cases (21.36%). Additionally, 2017 (34 cases, 10.53%), 2018 (38 cases, 11.76%), and 2024 (47 cases, 14.55%) had relatively higher proportions. Other years contributed to the dataset but with smaller percentages, as detailed in the baseline table. In FAERS, most reports were concentrated in 2009, with 870 cases (44.16%), and 2010, with 689 cases (34.97%). A few cases were reported each year from 2011 to 2024. Overall, WHO-VigiAccess does not exhibit a clear trend in reporting proportions, whereas FAERS shows a general decline in adverse event reports since 2009. This highlights a significant difference in the temporal distribution of reports between the two databases. Regarding geographical distribution, WHO-VigiAccess reports were primarily from Asia (210 cases, 65.02%) and the Americas (110 cases, 34.06%). In contrast, nearly all FAERS reports originated from North America (1,917 cases, 97.31%). These findings suggest that WHO-VigiAccess data may be more applicable to Asian countries, while FAERS data is more relevant to North American populations. By integrating both databases, this study provides a more comprehensive evaluation of the real-world safety of fenofibric acid across different regions.

Due to strict data protection laws and related agreements, WHO-VigiAccess data only includes statistical distributions based on gender, age, year, and region. In contrast, for FAERS data, we conducted a more detailed statistical analysis, further examining the distribution of adverse event reports by reporting country, reporter type, and severity. For example: the main reporters were consumers (613, 31.12%) and physicians (1,033, 52.44%). The majority of adverse event reports originated from the United States, with 1,893 cases (96.09%). A total of 1,632 cases (82.84%) were classified as non-serious. It is important to note that the classification of seriousness in this context is based on the patient dimension rather than the specific adverse event. In the FAERS database, cases with reported outcomes are considered serious, while those without reported outcomes are considered non-serious. For more detailed information and results, please refer to Supplementary Table 3.

### 3.2 Distribution of adverse events at the SOC level

**FIGURE 1:**
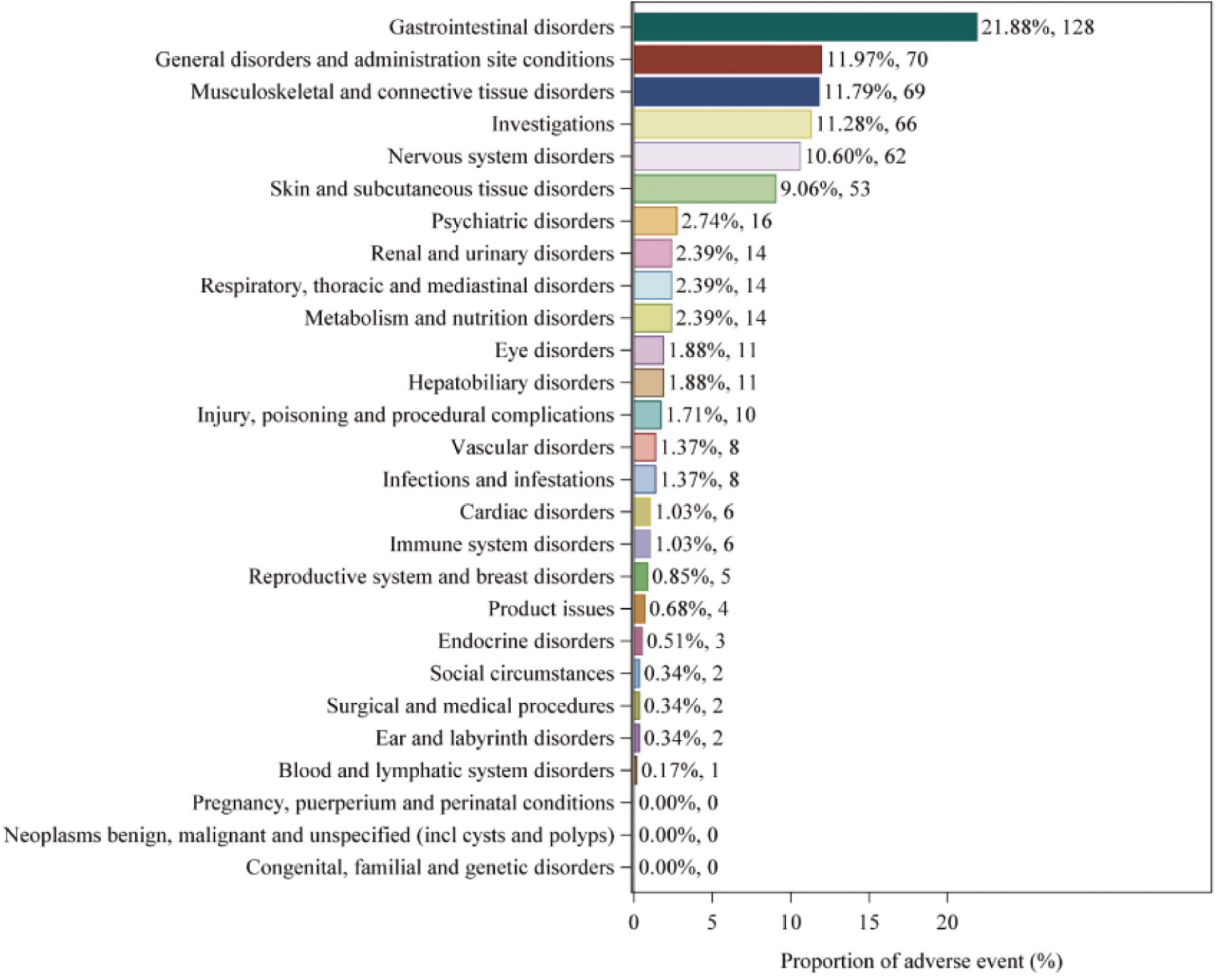
Distribution of adverse events in VigiAccess data at SOC level. The number of fenofibric acid-induced ADEs at the SOC level in VigiAccess. Percentage values represent the proportion of cases with such ADEs out of the total reported ADEs. SOC, System Organ Class; ADE, adverse drug event; VigiAccess, WHO Global Individual Case Safety Reports Database Access.

**FIGURE 2:**
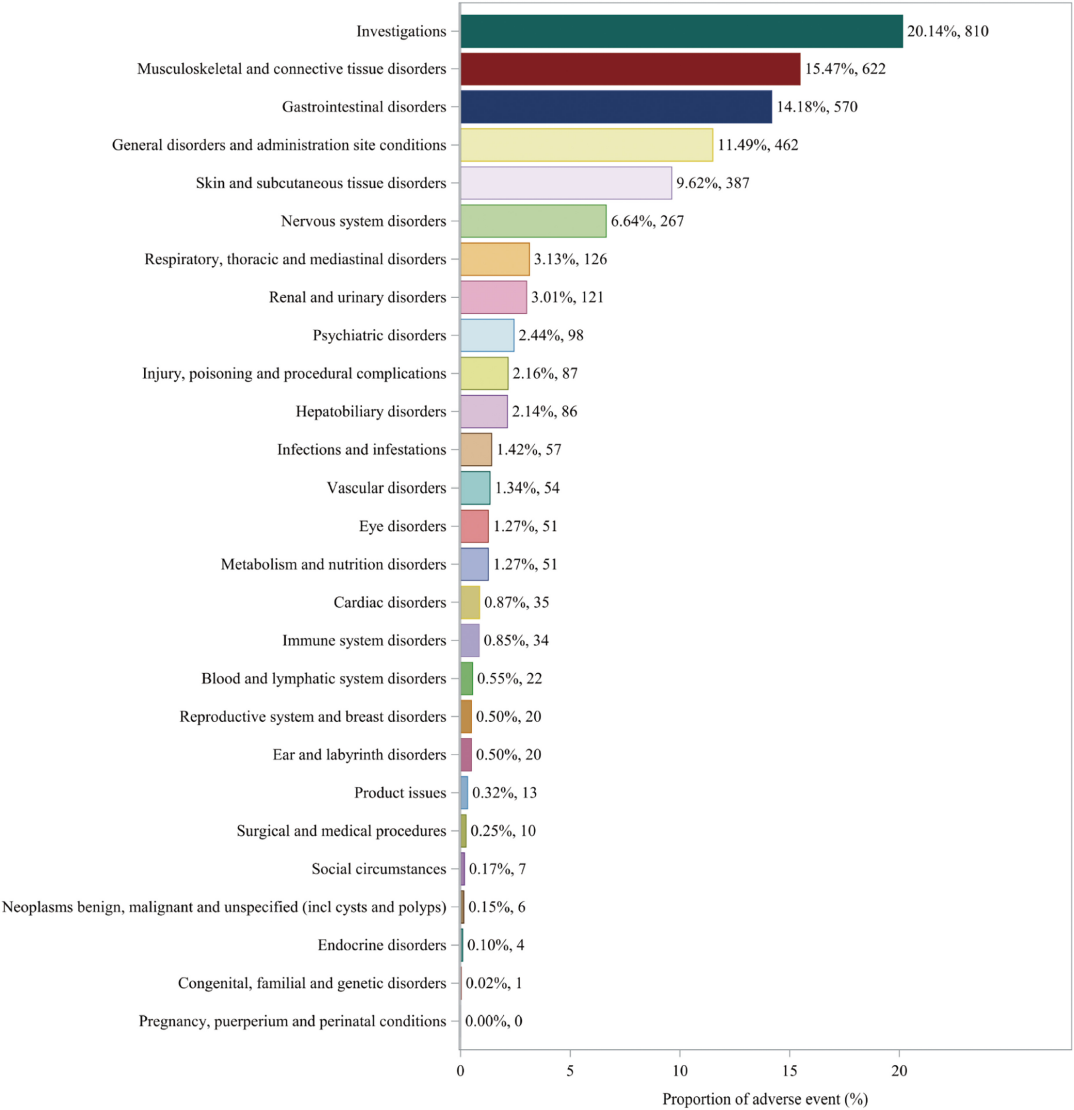
Distribution of adverse events in FAERS data at SOC level. The number of fenofibric acid-induced ADEs at the SOC level in FAERS. Percentage values represent the proportion of cases with such ADEs out of the total reported ADEs. SOC, System Organ Class; ADE, adverse drug event; FAERS, FDA Adverse Event Reporting System.

Adverse events associated with fenofibric acid were reported in 24 out of 27 SOCs in VigiAccess, with detailed information available in Supplementary Table 4. In FAERS, there are 26 types of SOCs. In VigiAccess and FAERS, the distribution of adverse events at the SOC level is illustrated in Figures 1, 2. It was observed that in VigiAccess, the top three SOCs were gastrointestinal disorders, general disorders and administration site conditions, and musculoskeletal and connective tissue disorders. In FAERS, the top three SOCs were investigations, musculoskeletal and connective tissue disorders, and gastrointestinal disorders.

Additionally, further analysis was conducted on FAERS data to identify statistically significant positive signals at the SOC level. Analysis of the distribution of positive signals for fenofibric acid-related adverse events across different SOCs identified several statistically significant signals. These included investigations, musculoskeletal and connective tissue disorders, gastrointestinal disorders, general disorders and administration site conditions, skin and subcutaneous tissue disorders, renal and urinary disorders, hepatobiliary disorders, and metabolism and nutrition disorders. Detailed analysis results can be found in Supplementary Table 5.

### 3.3 Distribution of adverse events at the PT level

**FIGURE 3:**
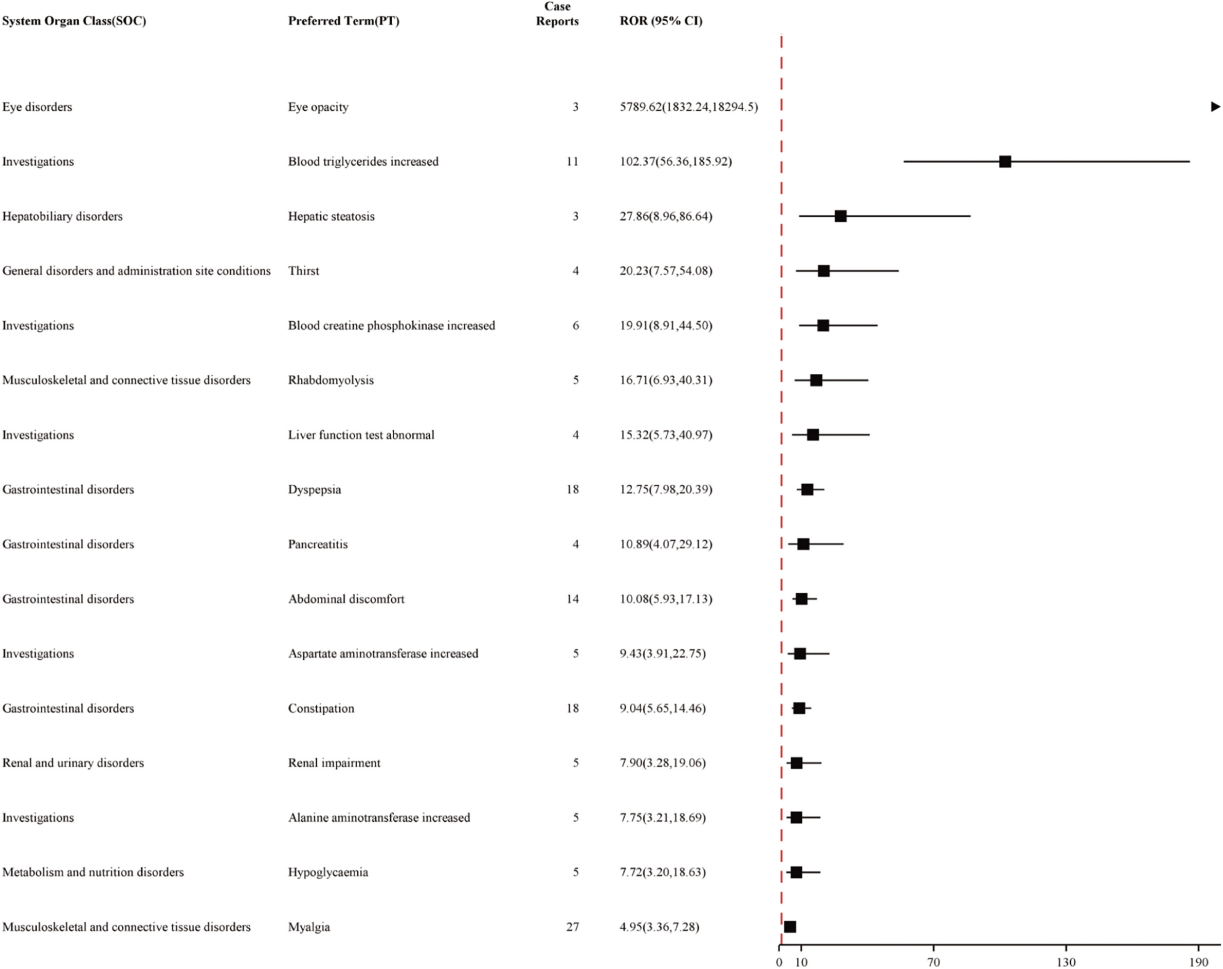
Top 16 Signal strength of AEs at PT level ranked by ROR of VigiAccess data. Figure illustrating the top 16 PT statistics in VigiAccess. The figure shows the SOC type of each PT, as well as its quantity and ROR value along with their confidence intervals. The PTs listed in the figure are ordered by ROR value. AE, adverse event; ROR, Reporting Odds Ratio; SOC, System Organ Class; PT, preferred term; VigiAccess, WHO Global Individual Case Safety Reports Database Access.

**FIGURE 4:**
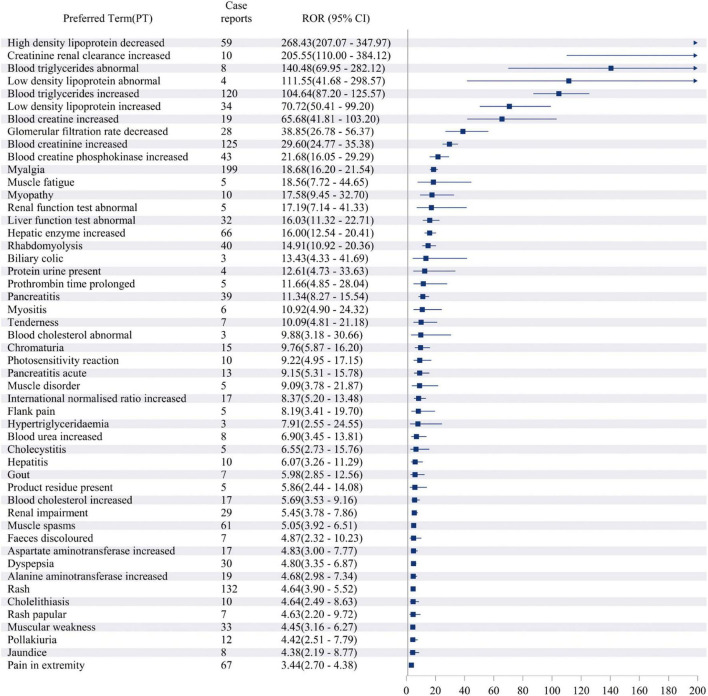
Top 50 Signal strength of AEs at PT level ranked by ROR of FAERS data. Figure illustrating the top 50 PT statistics in FAERS. The figure shows the PTs’ quantity and ROR value along with their confidence intervals. The PTs listed in the figure are ordered by ROR value. AE, adverse event; ROR, Reporting Odds Ratio; PT, preferred term; FAERS, FDA Adverse Event Reporting System.

The study analyzed all adverse event reports and signals at the PT level, with a particular focus on the top 50 most frequently reported and strongest signals in the VigiAccess and FAERS databases. At the PT level of VigiAccess and FAERS databases, detailed information on positive signals related to fenofibric acid, ranked by ROR values, is presented in Figures 3, 4, respectively. We found that, aside from the adverse reactions already mentioned in the drug label, eye opacity had the strongest signal in VigiAccess. In FAERS, myalgia was the most significant adverse event within the musculoskeletal and connective tissue disorders category. Notably, myalgia was also identified in VigiAccess, suggesting it may have a strong association with adverse reactions to fenofibric acid. Additionally, blood creatine phosphokinase increased appeared in the analysis of both databases, pointing to a potential adverse reaction and aligning with the previously mentioned concern about muscle injury. Regarding gastrointestinal disorders, dyspepsia had the strongest signal in VigiAccess and was also noted in the FAERS analysis. This implies that the drug may have certain adverse effects on the digestive system.

These analytical results are used to assess the positive signals of fenofibric acid-associated adverse events at the PT level. In addition to the currently known adverse reactions related to fenofibric acid, such as renal injury, hepatobiliary toxicity, pancreatitis, and allergic reactions. Our study also found several potential adverse reactions associated with fenofibric acid, including gout, hypoglycemia, prothrombin time prolonged, photosensitivity reactions, rash, blood creatine and creatinine increased, blood creatine phosphokinase increased, myalgia, muscle fatigue, pain in extremity, joint pain and headache. These adverse reactions need to be verified further clinical trials. Although gout, prothrombin time prolonged, photosensitivity reactions, and rash were not reflected in the VigiAccess analysis based on signal strength ranking, they have been observed and mentioned in some clinical trials of the drug. Supplementary Table 6 presents the top 16 adverse events in VigiAccess ranked by signal strength, while Supplementary Table 7 lists the top 50 adverse events in FAERS based on the same criteria. Due to the smaller dataset in VigiAccess compared to FAERS, only 16 PTs met the predefined thresholds (meta ≥ 3, PRR ≥ 2, Chi-Square ≥ 4, lower limit of 95% CI of ROR > 1, IC025 > 0, EBGM05 > 2) for inclusion in the analysis.

### 3.4 Time to onset and Weibull distribution analysis of adverse events

**FIGURE 5:**
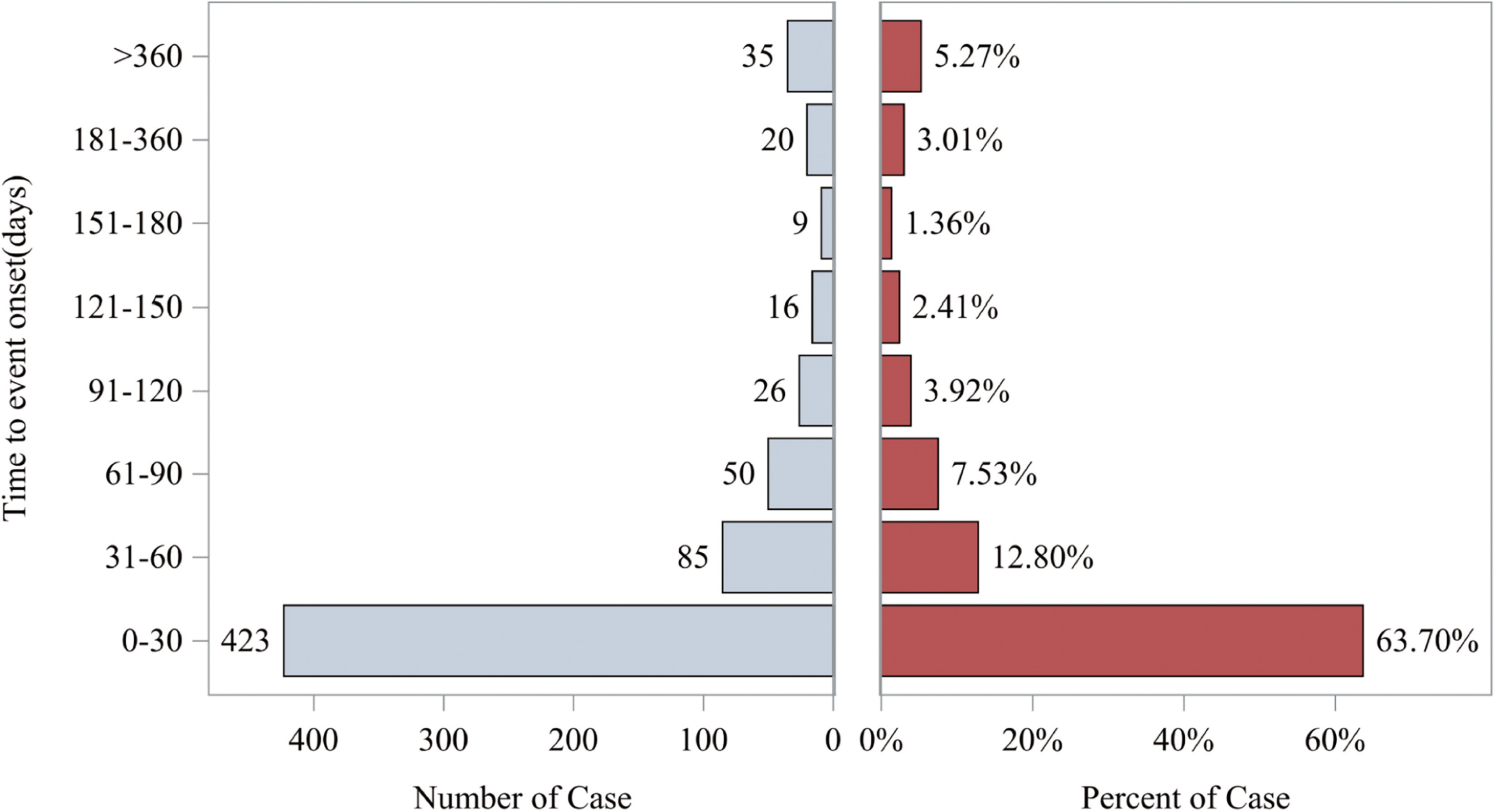
Time to event report distribution of AE reports. The left horizontal axis represents the number of cases occurring during different time periods. The right horizontal axis represents the percentage of cases in each time period relative to the total number of cases. AE, adverse event.

**FIGURE 6:**
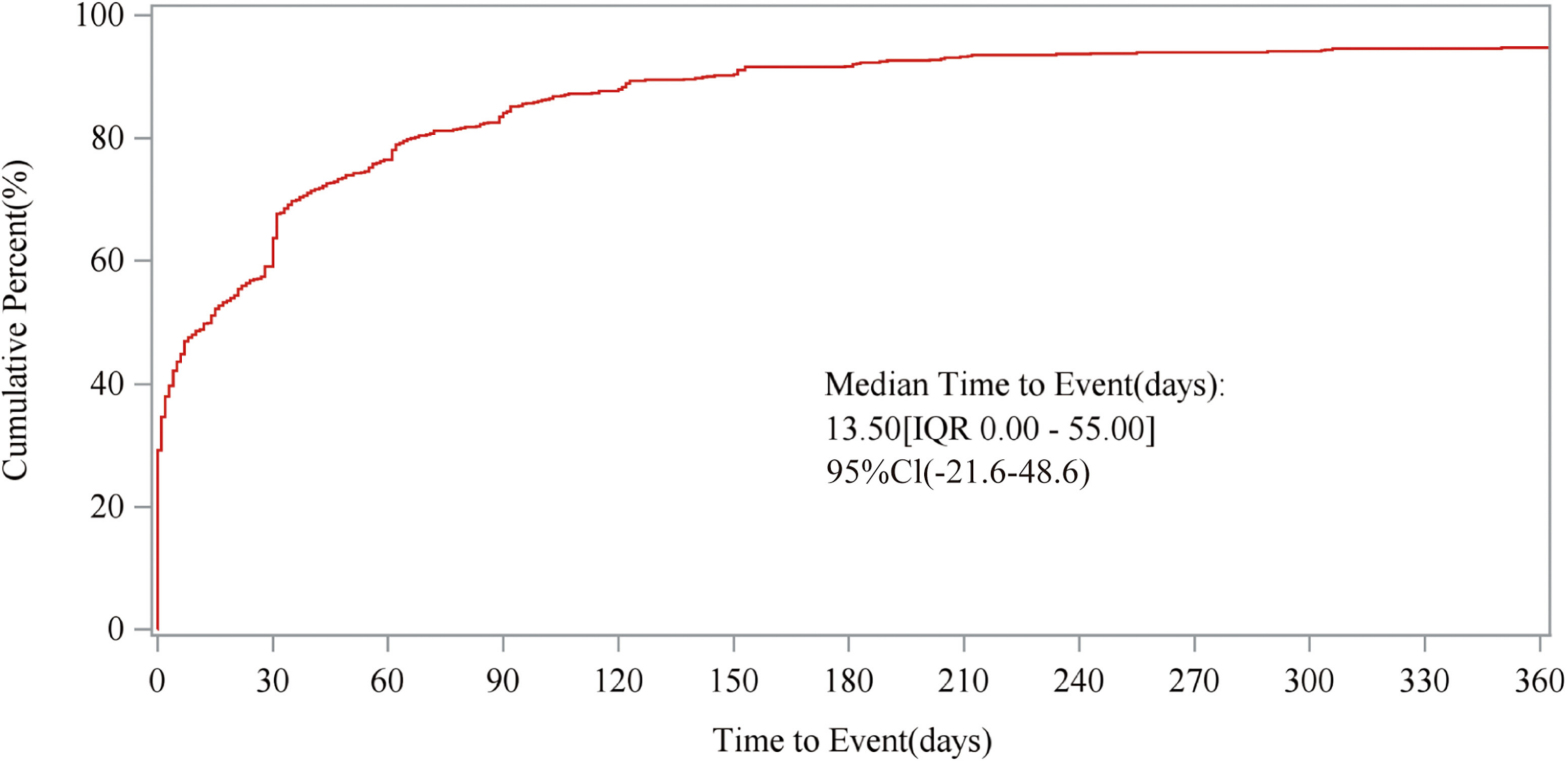
Cumulative incidence of adverse events. The figure illustrates the relationship between time to event and cumulative percent, presenting the median time to AE occurrence, IQR, and 95CI. AE, adverse event; IQR, interquartile range; 95CI, 95% confidence interval.

The study also analyzed the relationship between time and cumulative incidence based on data from the FAERS database. This study defines the occurrence time of fenofibric acid-related adverse events as the time difference between the adverse event occurrence date and the drug administration date. As shown in Figure 5, the number of reported cases in the first month was *n* = 423 (63.70%), in the second month *n* = 85 (12.80%), and in the third month *n* = 50 (7.53%). The majority of fenofibric acid-related adverse events were reported within the first 3 months, with a smaller number of cases occurring in the following 4 months and up to 1 year. The estimated Weibull shape parameter (β) was 0.68 (95% CI: 0.25–1.11), indicating an early failure type distribution, in which most fenofibric acid-related adverse events occurred shortly after treatment initiation and the risk decreased over time. Therefore, adverse events related to fenofibric acid primarily appear to occur within the first 3 months of treatment, though the possibility of adverse reactions occurring at later stages remains a possibility. Figure 6 illustrates the cumulative incidence of fenofibric acid-related adverse events. The 95% confidence interval for the median time to event was −21.6 to 48.6 days. Negative values arise from the normal-approximation method and indicate uncertainty near the lower boundary (true values cannot be negative). These findings provide a basis for monitoring and preventing fenofibric acid-related adverse reactions over time.

### 3.5 Analysis of gender differences in adverse events

**FIGURE 7:**
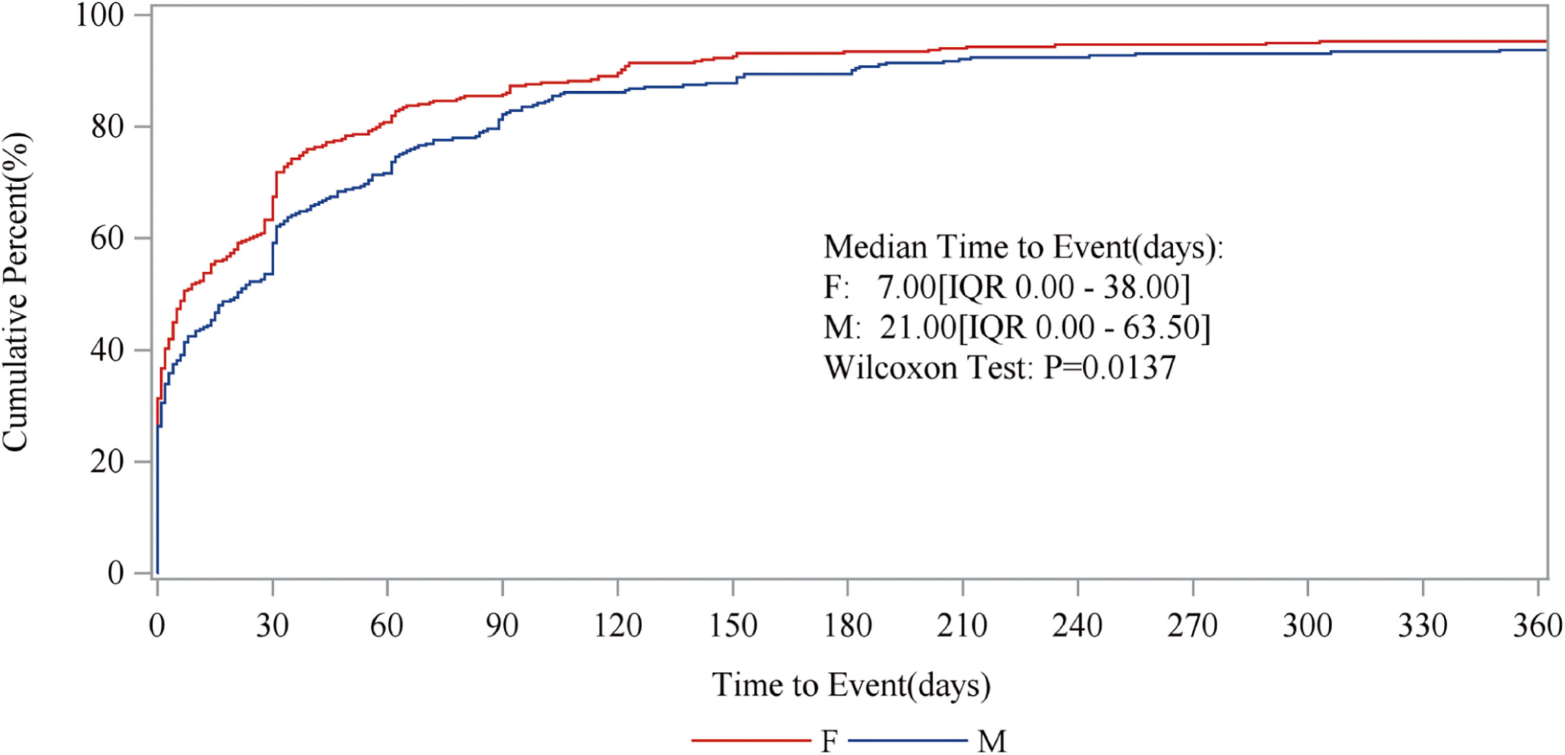
The relationship between gender and the cumulative incidence of adverse events. The red line represents the results of the female data analysis, while the blue line represents the male. The figure displays the median time to occurrence of adverse events for males and females, respectively, along with the *P*-values.

The study stratified FAERS reports by gender and examined the relationship between gender and the cumulative incidence of adverse events. Figure 7 presents the results, showing that the median time to event for females was 7 days (IQR 0.00–38.00), while for males, it was 21 days (IQR 0.00–63.50). This suggests that females may experience adverse events within a shorter timeframe. Additionally, the cumulative incidence curve for females rises more rapidly in the early stages, indicating a higher incidence of adverse events in women over the same period. The Wilcoxon test yielded a *P*-value of 0.0137, demonstrating that the difference in cumulative incidence between genders is statistically significant. Considering these findings, paying closer attention to adverse events in female patients may have important clinical implications when prescribing fenofibric acid. However, these findings require more precise trials to substantiate, as confounding factors were not eliminated in this gender differences study. This limitation will be discussed in detail in the subsequent discussion section.

## 4 Discussion

After the approval of fenofibrate for market use, the following adverse reactions have been identified: renal injury, renal failure, hepatitis, cirrhosis, cholelithiasis, pancreatitis, muscle spasms, rhabdomyolysis, anemia, fatigue, and interstitial lung disease, among others. Since fenofibrate is metabolized in the human body into fenofibric acid, which exerts its therapeutic effects, the adverse reactions observed with fenofibrate have also been noted with fenofibric acid. By applying the four disproportionality analysis methods to analyze relevant data from the WHO- VigiAccess and FAERS database (including adverse event reports associated with fenofibric acid from the first quarter of 2004 to the third quarter of 2024, covering a total of 83 quarters), we have observed some potential adverse reactions specific to fenofibric acid that distinguish it from fenofibrate: gout, hypoglycemia, prothrombin time prolonged, photosensitivity reactions, rash, blood creatine and creatinine increased, blood creatine phosphokinase increased, myalgia, muscle fatigue, pain in extremity, joint pain and headache. Pre-market clinical studies are rigorous and essential, serving as a key method for understanding drug adverse reactions. However, these studies are conducted under a set of controlled conditions, such as specific regions and environments, which may limit their ability to capture the full spectrum of adverse reactions among real-world drug users. As a result, they often lack a comprehensive assessment of a drug’s real-world safety. In contrast, spontaneous reporting systems (SRS) collect adverse event reports from patients after drug use, providing a more accurate reflection of real-world medication safety. Commonly used databases for real-world drug safety signal research include the Food and Drug Administration (FDA) Adverse Event Reporting System (FAERS), WHO-VigiBase, and the Eudra Vigilance Data Analysis System (EVDAS) (26). By integrating data from the WHO-VigiAccess and FAERS databases and analyzing the high incidence and strong signal intensity of common adverse reactions, this study provides a more comprehensive and precise clinical assessment of the real-world safety of fenofibric acid.

Further research and discussion are needed on the clinical safety and adverse reactions of fenofibric acid treatment. Additionally, through Weibull distribution analysis, we explored the relationship between fenofibric acid-related adverse reactions and time, with a particular focus on monitoring, mitigating, and preventing adverse reactions occurring within the first 3 months of fenofibric acid treatment for hyperlipidemia.

Although hypoglycemia was not identified in the FAERS data analysis, it exhibited a strong signal in the WHO-VigiAccess statistical results. Therefore, considering it as a potential adverse reaction is meaningful. Common pathological factors contributing to hypoglycemia include: insulinoma (excessive insulin secretion by pancreatic β-cells leading to decreased blood sugar levels), renal insufficiency (reduced renal clearance of insulin), liver dysfunction (impaired gluconeogenesis), endocrine disorders such as adrenal insufficiency and pituitary insufficiency, among others (27–29). These findings raise the possibility that the adverse reactions listed on the fenofibric acid drug label, such as drug-induced kidney injury and hepatotoxicity, may indirectly contribute to hypoglycemia. Additionally, improper drug use could also contribute to hypoglycemia, particularly when used alongside insulin or insulin analogs and sulfonylurea hypoglycemic agents (30). What we discovered is the potential relationship between fenofibric acid, a drug used to treat hyperlipidemia, and hypoglycemia. One study mentioned that through simulation-based methods, the authors identified fenofibric acid as a potential free fatty acid receptor 1 (FFA1) agonist. FFA1 is known to stimulate pancreatic β-cells to secrete insulin, which could explain its possible link to hypoglycemia (31). Collectively, after taking fenofibrate, it may be possible to increase the insulin level in the blood by activating FFA1, which may contribute to hypoglycemia as an adverse reaction. However, this hypothesis is based on computational modeling and has not yet been confirmed through clinical studies. Further research is needed to explore the precise interaction between fenofibric acid and hypoglycemia. Nonetheless, this potential mechanism offers a basis for clinicians to monitor the blood glucose levels of patients taking fenofibric acid, helping to prevent hypoglycemia from affecting patients’ daily lives while ensuring the drug’s therapeutic efficacy.

The adverse reactions discussed below—gout, prothrombin time prolonged, photosensitivity reactions, and rash—were not reflected in the statistical analysis results of VigiAccess. However, multiple large clinical trials have reported their occurrence. Therefore, we will also explore in detail the potential relationship between these adverse reactions and fenofibric acid.

Gout emerged as a significant signal associated with fenofibric acid in our analysis. Gout is primarily caused by the deposition of uric acid crystals in joints or non-joint structures, with hyperuricemia being a significant risk factor for its occurrence (32). The elevation of uric acid levels in the blood may result from two main mechanisms: disturbances in purine metabolism leading to increased uric acid production or impaired renal function causing uric acid excretion disorders (33). Studies have shown that renal injury is a known adverse reaction of fenofibric acid (34). A reasonable explanation for this could be that fenofibric acid-induced renal damage may lead to impaired uric acid excretion, which, over time, could promote the accumulation of uric acid crystals and contribute to the development of gout. Another possibility is the interaction between the drug’s target, PPARα, and urate transporter 1, which could influence uric acid excretion and reabsorption. The relationship between fenofibric acid and gout requires further investigation to be fully understood and substantiated. However, this potential association offers clinicians a basis for monitoring uric acid levels and preventing gout when prescribing fenofibric acid.

Prolonged prothrombin time appears to be strongly associated with fenofibric acid. Prothrombin time is a crucial indicator of the extrinsic coagulation pathway and is used to assess blood clotting ability. One possible reason for prolonged prothrombin time is an underlying liver disease, which may contribute to insufficient synthesis of certain coagulation factors, thereby extending prothrombin time. Collectively, one possible mechanism is the indirect effect of hepatotoxicity of the drug itself. Another possible explanation is drug interactions. In one study, researchers administered warfarin (2 mg/kg) alone or in combination with fenofibric acid (100 mg/kg) to rats. They found that the prothrombin time in the co-administration group was 199 ± 33 s, which was ten times longer than that in rats receiving warfarin alone. Further investigation revealed that this effect was due to fenofibric acid inhibiting warfarin metabolism (35). Collectively, in patients taking anticoagulant medications, fenofibric acid may alter certain pharmacological properties of these drugs, potentially increasing the risk of bleeding. Future studies are needed to explore the relationship between fenofibric acid and prolonged prothrombin time. Additionally, it has been reported in the literature that the efficacy of different anticoagulant drugs varies among different regions. For instance, warfarin is not suitable for anticoagulant therapy in northern China (36). This raises the possibility that fenofibric acid may also exhibit regional differences in the side effect of prolonged prothrombin time. Clinicians should advise patients undergoing fenofibric acid treatment to be cautious of this potential adverse reaction.

Photosensitivity reactions and rash are also positive signals that have strong correlation with fenofibric acid found through the research results. Photosensitivity reactions refers to an abnormal reaction of the skin to ultraviolet or visible light, which can be caused by various factors such as drugs, plants, and chemicals. In this study, we primarily refer to drug-induced photosensitivity (DIP), including phototoxic reactions and photoallergic reactions (37). In phototoxic reactions, the drug absorbs energy from ultraviolet light and releases it into the skin, causing damage. The main symptoms are erythema and edema. In photoallergic reactions, light may cause structural changes in the drug, forming a semi-antigen that binds with skin proteins to form a complex, leading to hypersensitivity reactions. Clinically, this manifests as papules, eczematous rashes, and urticarial-like rashes (38, 39). Drug-induced rashes generally arise from drug allergic reactions, drug photosensitivity reactions, direct drug irritation or toxicity, and other factors. Therefore, fenofibric acid may induce photosensitivity reactions after administration by interacting with ultraviolet light (altering its own structure or absorbing energy that damages cells), potentially resulting in erythema and papules in patients. This also indirectly supports the mechanism behind the occurrence of papules as a potential adverse reaction. The cause of papule formation may also be related to the prolonged effect of fenofibric acid, potentially resulting in fatty acid metabolism disorders, lipotoxic damage to cell membranes and organelles, mitochondrial dysfunction, the generation of reactive oxygen species, and local inflammatory responses, all contributing to the development of papules. However, the relationship between fenofibric acid and photosensitivity reactions and papules still requires further clinical trials for validation. In conclusion, photosensitivity reactions and papules are potential adverse reactions found in this study with strong correlation with fenoflavinic acid, independent of the post-marketing adverse reactions of fenofibrate. For patients experiencing these adverse reactions while using fenofibric acid, it may be considered to switch to fenofibrate as an alternative treatment in the future. This provides guidance for the clinical safe use of fenofibric acid.

Blood creatine phosphokinase increased and myalgia were observed in both databases with strong signal intensity, suggesting that they may be potential adverse reactions associated with fenofibric acid.

Blood creatine and creatinine increased, blood creatine phosphokinase increased, myalgia, and muscle fatigue have been found to be positive signals with higher signal intensity. In a clinical study investigating the effects of fenofibric acid alone and in combination with statins on patients with dyslipidemia, it was found that an adverse event occurring in ≥ 3% of patients in any treatment group was elevated blood creatine kinase (CK). Compared to 2 cases (0.5%) in the low-dose statin group, there were 8 cases (2%) in the fenofibric acid + low-dose statin group (40). Additionally, in this study, when comparing the fenofibric acid + low-dose statin and fenofibric acid + moderate-dose statin groups with the low-dose statin and moderate-dose statin groups, increases were observed in the three indicators: creatinine > 2 mg/dL, creatinine > 2 × baseline, and creatinine > 1.5 × baseline and above upper limit of normal (ULN) (40). Furthermore, other studies have found that the transient increase in serum creatinine induced by fenofibric treatment is reversible (41, 42). Based on the data, fenofibric acid is highly likely to cause myopathy-related adverse reactions, and these effects are likely reversible after discontinuation of the drug. However, further clinical studies and pathophysiological research are needed to explore and elucidate the relationship between fenofibric acid and myopathy.

We all know that myopathy refers to primary structural or functional abnormalities of the muscles, primarily affecting skeletal muscle. It often includes symptoms such as myalgia, rhabdomyolysis, muscle weakness, and muscle atrophy, which may lead to muscle tissue breakdown and necrosis, as well as the release of intracellular components into the bloodstream, resulting in elevated serum creatine, creatinine, and creatine phosphokinase levels (43). Analyzing the relationship between fenofibric acid and myopathy from its target perspective, one of its primary targets, peroxisome proliferator-activated receptors (PPARs), consists of three subtypes: PPARα, PPAR-β/δ, and PPAR-γ. These subtypes are, respectively, involved in efficient fatty acid catabolism, energy metabolism, mitochondrial biogenesis and fiber type transformation, as well as lipid deposition in muscles and other organs (44, 45). Based on these functions, PPARα does not seem to be directly associated with myopathy and may even have a protective role. However, many clinical studies on fenofibric acid have reported adverse reactions such as myalgia and muscle fatigue (40, 46–48). In some cases, severe adverse events such as rhabdomyolysis have been observed (49). These findings suggest that fenofibric acid itself may not directly cause severe myopathy but rather exerts its effects through PPAR activation. The most plausible hypothesis is that prolonged use of fenofibric acid contributes to excessive activation of PPARα, disrupting muscle fatty acid metabolism and mitochondrial function, ultimately damaging muscle tissue. Additionally, activation of other PPAR subtypes may contribute to lipid accumulation and muscle fiber transformation. Inhibition of L-FABP could also lead to lipid deposition in muscle tissue. These mechanisms may ultimately result in muscle degeneration and necrosis, with the release of intracellular contents causing elevated serum creatine, creatinine, and creatine phosphokinase levels, as well as symptoms such as myalgia and muscle fatigue. This, in turn, supports the previously mentioned potential adverse reactions and the reversible nature of creatinine elevation. Whether this hypothesis is correct requires further research to confirm the relationship between fenofibric acid and myopathy. Nevertheless, these findings provide healthcare professionals with valuable insights into monitoring and addressing muscle-related adverse effects in patients undergoing fenofibric acid treatment.

Our study results also suggest that potential adverse reactions of fenofibric acid include pain in extremity, joint pain, headache, and gastrointestinal-related adverse effects. Several studies on fenofibric acid have reported headache as an adverse reaction (40, 46, 47, 50). Skeletal muscle atrophy occurs in many disease processes and is often accompanied by lipid deposition in muscle tissue and mild chronic inflammation (44). Therefore, during the aging and damage process of muscle tissue, joint pain and extremity pain may occur due to increased motor impairment, or they may result from an underlying gout condition in patients. This aligns with our previous discussion on the relationship between fenofibric acid, gout, and muscle-related adverse reactions. Gastrointestinal-related adverse effects are also worth attention and prevention, as they may be associated with treatment interruption and patient aversion to therapy. Of course, the discussion of the above adverse reactions is only at the level of guessing. In the future, specific experiments are needed to explore the relationship between the two. Additionally, some studies have found that adverse reactions such as non-cardiogenic chest pain, epigastric pain, dyspepsia, and esophageal pain may also be related to the drug (47).

The potential adverse reactions strongly associated with fenofibric acid identified in this study also appear to exhibit regional differences. As mentioned earlier, WHO-VigiAccess data are more representative of Asian populations, whereas FAERS data primarily reflect North American populations. Accordingly, we speculate that the strong signal for hypoglycemia observed in VigiAccess may be more common among Asian patients, while gout, prothrombin time prolonged, photosensitivity reactions, and rash, which showed strong signals in FAERS, may be more prevalent in American populations. The adverse events showing positive signals in both databases, such as blood creatine phosphokinase increased and myalgia, are likely to be common across all populations. These regional variations in potential adverse reactions may result from multiple intertwined factors, including differences in genetic backgrounds, medication habits, drug-metabolizing enzyme diversity, prescribing preferences, and the prevalence of comorbidities among populations. Therefore, considering regional heterogeneity is essential when evaluating the adverse reactions associated with fenofibric acid.

We performed a Weibull distribution analysis on FAERS data and predicted the timing and cumulative incidence of adverse reactions associated with fenofibric acid as the primary suspected drug. It suggests that we should monitor and prevent the occurrence of these adverse events within the 3 months following fenofibric acid treatment. This provides a basis for preventing adverse reactions over time and helps to reduce the impact of these reactions on patients’ lives, thereby improving their quality of life. Additionally, based on gender-stratified analysis, we found that female patients may have a higher incidence of adverse events after taking the drug. There are multiple factors contributing to the occurrence of sex-biased adverse events (SBAEs). According to the literature, the underlying causes of SBAEs include, but are not limited to, differences in body weight, hormonal levels, pharmacokinetics, hepatic drug metabolism, and transporter activity (51). Among these factors, sex hormones are hypothesized to influence SBAEs by competing for drug transporters, interacting with metabolic enzymes, or inhibiting enzymatic activity (52). Recent studies have also revealed that many SBAEs are associated with sex-specific differences in the expression and regulation of drug targets and drug-metabolizing genes (53). Therefore, based on both current findings and previous reports, enhanced monitoring of adverse drug reactions among female patients is of important clinical significance.

Study also has certain limitations. The description of patients’ age distribution in the manuscript may not be entirely accurate because part of the age information is missing in both datasets. Therefore, additional and more comprehensive data are needed to provide a more reliable characterization of the age distribution. In terms of data sources, VigiAccess data primarily comes from Asian countries, making its statistical analysis more applicable to Asian populations. Similarly, FAERS data predominantly originates from the Americas, meaning its results are more relevant to American populations. This geographic imbalance may lead to regional bias in the detected safety signals, as variations in reporting habits, healthcare systems, and drug usage patterns across regions can influence the frequency and type of adverse events reported. The overlap of significant adverse reactions between the two databases is relatively small, highlighting the importance of considering regional differences when evaluating drug-related adverse events.

In addition, both FAERS and VigiAccess databases are subject to inherent limitations of SRS, including underreporting, duplicate entries, and potential misclassification of drugs or adverse events. These factors may distort the true distribution or strength of the observed safety signals, as some adverse reactions might be underrepresented or incorrectly linked to the suspected drug. To mitigate these limitations, we screened the data during inclusion to eliminate duplicates and select only those records where the fenofibric acid was the primary suspect medication. Furthermore, some data is sourced from consumer reports, which may lead to inaccuracies or missing key information, potentially causing deviations in the results of this study.

Another limitation of this study is that potential confounding factors were not controlled for in the gender-stratified analysis. Differences in age distribution between male and female patients may have influenced the observed timing of adverse events, as older individuals often have decreased drug metabolism and altered pharmacokinetics, which could lead to earlier or more frequent adverse reactions. In addition, variations in comorbidities such as diabetes, hypertension, or renal impairment may have contributed to the observed differences, since these conditions can increase susceptibility to drug-related events and modify drug clearance. Moreover, polypharmacy—the concurrent use of multiple medications—tends to be more common in certain subgroups and may amplify drug–drug interactions, further affecting the onset and severity of adverse events. Because the FAERS and VigiAccess databases do not provide detailed clinical or medication history information, these confounding factors could not be adjusted for in the current analysis. Therefore, the earlier onset of adverse events observed in female patients should be interpreted with caution, as it may partly reflect underlying demographic or clinical differences rather than a true sex-related effect.

In terms of data analysis, this study solely relied on data analysis to draw conclusions and discuss findings, lacking clinical trial support, which may introduce bias. All signal detection results only indicate statistical associations and represent the relative magnitude of risk; they do not establish causality or quantify absolute risk. Therefore, the safety signals identified in this study should be interpreted cautiously, considering the above-mentioned limitations of data quality and regional coverage. Meanwhile, these data cannot be used to calculate the incidence rate. To reduce errors, future research should involve large datasets from more diverse regions and professional populations for analysis, and further clinical trials are needed to validate the safety of fenofibric acid.

## 5 Conclusion

This study utilized the four disproportionality analysis methods to analyze data from VigiAccess and FAERS, where fenofibric acid was the primary suspect drug. The results validated known adverse reactions of fenofibric acid, such as kidney injury, hepatobiliary toxicity, pancreatitis, and allergic reactions, while also finding some potential adverse reactions with strong correlation with fenofibric acid, such as gout, hypoglycemia, prothrombin time prolonged, photosensitivity reactions, rash, blood creatine and creatinine increased, blood creatine phosphokinase increased, myalgia, muscle fatigue, pain in extremity, joint pain and headache. It also highlights the importance of monitoring patients for adverse reactions within the first 3 months. The study also indicates that increased attention to adverse events in female patients may be clinically meaningful. This provides doctors with more safety information regarding fenofibric acid.

## Data Availability

The datasets presented in this study can be found in online repositories. The names of the repository/repositories and accession number(s) can be found at: the original contributions presented in the study are included in the article/Supplementary material, further inquiries can be directed to the corresponding authors. The raw data of WHO-Vigiaccess can be downloaded from https://www.vigiaccess.org. The raw data of FAERS can be downloaded from https://fis.fda.gov/extensions/FPD-QDE-FAERS/FPD-QDE-FAERS.html.
