# Supplementary material for: Assessing the real-world safety of fenofibric acid for hyperlipidemia: results from WHO-VigiAccess and FAERS databases
Source: Front Med (Lausanne). 2025 Nov 7;12:1702197. doi: 10.3389/fmed.2025.1702197 (PMC12634654; doi:10.3389/fmed.2025.1702197)
Supplement: Supplementary file 1 [file Supplementary_file_1.docx]

Supplementary Material

**Supplementary Table 1** **Two-by-two contingency table for disproportionality analyses**

|  | **Target AEs** | **Other AEs** | **Total** |
| --- | --- | --- | --- |
| **Fenofibric acid** | a | b | a+b |
| **Other Drugs** | c | d | c+d |
| **Total** | a+c | b+d | N=a+b+c+d |

# Abbreviations: AEs, adverse events; a, the number of reports containing target AEs caused by fenofibric acid; b, the number of reports containing other AEs caused by fenofibric acid; c, the number of reports containing target AEs caused by other drugs; d, the number of reports containing other AEs caused by other drugs; N, the total number of adverse event occurrences included in the background data analysis.

**Supplementary Table 2** **Formulas and thresholds of disproportionality analysis methods**

| **Method** | **Formula** | **Threshold** |
| --- | --- | --- |
| **ROR** | 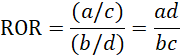  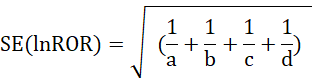  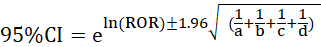 | a ≥ 3 and 95% CI (lower limit) > 1 suggests generation of 1 signal. |
| **PRR** | 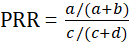  SE (lnPRR) =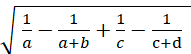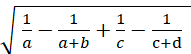  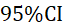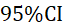=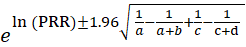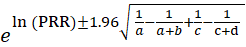  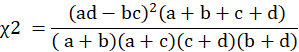 | Lower limit of the PRR confidence interval: a ≥ 3 and 95% CI (lower limit) > 1 suggests generation of 1 signal.  MHRA composite criterion method: a ≥ 3 and PRR value ≥ 2 and χ² ≥ 4 suggests generation of 1 signal |
| **BCPNN** | IC=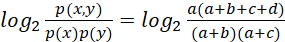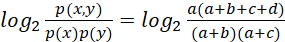  E(IC)=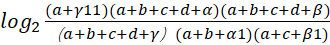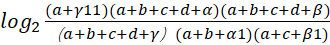  V(IC)=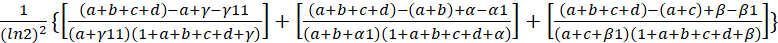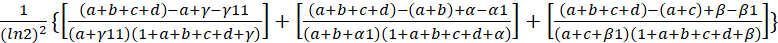  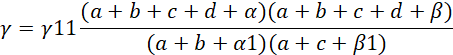  *IC-2SD=E(IC)-2*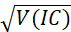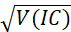  α1=β1=1；α=β=2；γ11=1 | The lower limit of the confidence interval (IC-2SD) is greater than 0, then 1 signal is prompted to be generated |
| **MGPS** | 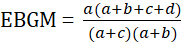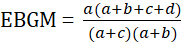  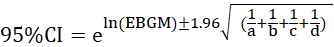 | EBGM05>2 prompts to generate 1 signal  EBGM05 indicates the lower limit of the EBGM 95% confidence interval |

Abbreviations: ROR, Reporting Odds Ratio; PRR, Proportional Reporting Ratio; BCPNN., Bayesian confidence propagation neural network; MGPS, Muti-item Gamma Poisson Shrinker; MHRA, Medicines and Health care Products Regulatory Agency. The results of the PRR lower confidence interval method for signal detection are almost the same as those of the ROR method, and the threshold of the MHRA composite standard method is commonly used for signal detection. In the summary report, the threshold of the MHRA composite standard method will be used as the threshold of the PRR method for signal detection.

**Supplementary Table 3** **Characteristics of AEs reports**

| **Characteristics** | Fenofibric acid | |
| --- | --- | --- |
|  | **VigiAccess(n=323)** | **FAERS(n=1970)** |
|  | **n (%)** | **n (%)** |
| Sex |  |  |
| Female | 155(47.99) | 748(37.97) |
| Male | 140(43.34) | 765(38.83) |
| Unknown | 28(8.67) | 457(23.20) |
| Age |  |  |
| <18 | 0(0) | 2(0.10) |
| 18 - 44 years | 24(7.43) | 61(3.10) |
| 45 - 64 years | 133(41.18) | 314(15.94) |
| 65 - 74 years | 64(19.81) | 208(10.56) |
| 75 years | 28(8.67) |  |
| Unknown | 74(22.91) | 1385(70.30) |
| Continent |  |  |
| Africa | 3(0.93) | 1(0.05) |
| Americas | 110(34.06) | 1917(97.31) |
| Asia | 210(65.02) | 2(0.10) |
| Europe | 0(0) | 6(0.30) |
| Not Specified | 0(0) | 44(2.23) |
| Report year |  |  |
| 2009 | 0(0) | 870(44.16) |
| 2010 | 3(0.93) | 689(34.97) |
| 2011 | 16(4.95) | 192(9.75) |
| 2012 | 4(1.24) | 91(4.62) |
| 2013 | 0(0) | 48(2.44) |
| 2014 | 9(2.79) | 17(0.86) |
| 2015 | 16(4.95) | 10(0.51) |
| 2016 | 6(1.86) | 10(0.51) |
| 2017 | 34(10.53) | 2(0.10) |
| 2018 | 38(11.76) | 6(0.30) |
| 2019 | 24(7.43) | 9(0.46) |
| 2020 | 69(21.36) | 5(0.25) |
| 2021 | 22(6.81) | 4(0.20) |
| 2022 | 4(1.24) | 4(0.20) |
| 2023 | 31(9.6) | 9(0.46) |
| 2024 | 47(14.55) | 4(0.20) |
| Reporter |  |  |
| Consumer |  | 613(31.12) |
| Lawyer |  | 1(0.05) |
| Not Specified |  | 54(2.74) |
| Other health-professional |  | 233(11.83) |
| Pharmacist |  | 36(1.83) |
| Physician |  | 1033(52.44) |
| Reporting countries |  |  |
| United States of America |  | 1893(96.09) |
| Puerto Rico |  | 20(1.02) |
| Germany |  | 3(0.15) |
| Mexico |  | 2(0.10) |
| United Kingdom |  | 2(0.10) |
| Severity |  |  |
| Non-Serious |  | 1632(82.84) |
| Serious |  | 338(17.16) |
| Outcome |  |  |
| Life-Threatening |  | 11(0.56) |
| Hospitalization - Initial or Prolonged |  | 129(6.55) |
| Disability |  | 10(0.51) |
| Death |  | 12(0.61) |
| Congenital Anomaly |  | 3(0.15) |
| Required Intervention to Prevent Permanent Impairment/Damage |  | 15(0.76) |
| Other |  | 223(11.32) |
| Timing of adverse events |  |  |
| 0-30d |  | 423(21.47) |
| 31-60d |  | 85(4.31) |
| 61-90d |  | 50(2.54) |
| 91-120d |  | 26(1.32) |
| 121-150d |  | 16(0.81) |
| 151-180d |  | 9(0.46) |
| 181-360d |  | 20(1.02) |
| ＞360d |  | 35(1.78) |
| Missing or outliers(＜0) |  | 1306(66.29) |
| Weight (KG) |  |  |
| N(Missing) |  | 707(1263) |
| Mean (SD) |  | 85.67(19.59) |
| Median (Q1, Q3) |  | 81.72(72.19,96.25) |
| Min, Max |  | 43.13,165.71 |

Note 1: The outcome is based on the patient dimension, not specific adverse events. The same patient may have multiple outcomes in the database, so the sum of outcomes may not equal 100%

Note 2: Serious reports are based on patient dimensions, not specific adverse events. In the FAERS database, serious reports are those with outcome information filled in, while non-serious reports are those without outcome information

Note 3: The date of adverse events collected by the database refers to the date when the patient first experienced an adverse event (without specifying the specific name of the adverse event). In calculating the time of adverse event occurrence - medication date (days), values less than 0 days are treated as missing values for statistical purposes. This type of data generally focuses more on the median.

**Supplementary Table 4** **Signal strength of ADEs at the System Organ Class (SOC) level**

| **SOC** | **Vigiaccess** | **FAERS** |
| --- | --- | --- |
|  | **Case reports** | **Case reports** |
| Gastrointestinal disorders | 128 | 570 |
| General disorders and administration site conditions | 70 | 462 |
| Musculoskeletal and connective tissue disorders | 69 | 622 |
| Investigations | 66 | 810 |
| Nervous system disorders | 62 | 267 |
| Skin and subcutaneous tissue disorders | 53 | 387 |
| Psychiatric disorders | 16 | 98 |
| Metabolism and nutrition disorders | 14 | 51 |
| Respiratory, thoracic and mediastinal disorders | 14 | 126 |
| Renal and urinary disorders | 14 | 121 |
| Hepatobiliary disorders | 11 | 86 |
| Eye disorders | 11 | 51 |
| Injury, poisoning and procedural complications | 10 | 87 |
| Infections and infestations | 8 | 57 |
| Vascular disorders | 8 | 54 |
| Immune system disorders | 6 | 34 |
| Cardiac disorders | 6 | 35 |
| Reproductive system and breast disorders | 5 | 20 |
| Product issues | 4 | 13 |
| Endocrine disorders | 3 | 4 |
| Ear and labyrinth disorders | 2 | 20 |
| Surgical and medical procedures | 2 | 10 |
| Social circumstances | 2 | 7 |
| Blood and lymphatic system disorders | 1 | 22 |
| Neoplasms benign, malignant and unspecified (incl cysts and polyps) | 0 | 6 |
| Congenital, familial and genetic disorders | 0 | 1 |

**Supplementary Table 5** **Distribution of positive signals of adverse events of target drugs in different SOCs**

| **SOC** | **Number of positive signals** | **Proportion (%)** |
| --- | --- | --- |
| Investigations | 22 | 40.00 |
| Musculoskeletal and connective tissue disorders | 11 | 20.00 |
| Gastrointestinal disorders | 6 | 10.91 |
| General disorders and administration site conditions | 2 | 3.64 |
| Skin and subcutaneous tissue disorders | 4 | 7.27 |
| Nervous system disorders | 0 | 0.00 |
| Respiratory, thoracic and mediastinal disorders | 0 | 0.00 |
| Renal and urinary disorders | 3 | 5.45 |
| Psychiatric disorders | 0 | 0.00 |
| Injury, poisoning and procedural complications | 0 | 0.00 |
| Hepatobiliary disorders | 5 | 9.09 |
| Infections and infestations | 0 | 0.00 |
| Vascular disorders | 0 | 0.00 |
| Metabolism and nutrition disorders | 2 | 3.64 |
| Eye disorders | 0 | 0.00 |
| Cardiac disorders | 0 | 0.00 |
| Immune system disorders | 0 | 0.00 |
| Blood and lymphatic system disorders | 0 | 0.00 |
| Ear and labyrinth disorders | 0 | 0.00 |
| Reproductive system and breast disorders | 0 | 0.00 |
| Product issues | 0 | 0.00 |
| Surgical and medical procedures | 0 | 0.00 |
| Social circumstances | 0 | 0.00 |
| Neoplasms benign, malignant and unspecified (incl cysts and polyps) | 0 | 0.00 |
| Endocrine disorders | 0 | 0.00 |
| Congenital, familial and genetic disorders | 0 | 0.00 |
| Pregnancy, puerperium and perinatal conditions | 0 | 0.00 |
| Total | 55 | 100.00 |

Note 1: the number of positive signals refers to the number of signals detected by the preferred term (PT) in the method organ system classification (SOC). Note that the number is the number of PT species, not the number of PT occurrences;

Note 2: Proportion of signals in SOC = Number of signals in SOC/total number of signals in target drug.

**Supplementary Table 6** **Signal strength of adverse events at the Preferred Term (PT) level ranked by ROR**

| **Preferred Term (PT)** | **Case reports** | **ROR (95% CI)** | **PRR (95% CI)** | **Chi Square** | **IC (IC025)** | **EBGM (EBGM05)** |
| --- | --- | --- | --- | --- | --- | --- |
| Eye opacity | 3 | 5789.62  (1832.24,18294.5) | 5759.94  (1833.33,18096.5) | 16794.0 | 12.45  (0.53) | 5599.97  (1772.22) |
| Blood triglycerides increased | 11 | 102.37  (56.36,185.92) | 100.46  (55.94,180.42) | 1082.84 | 6.65  (2.59) | 100.41  (55.29) |
| Hepatic steatosis | 3 | 27.86  (8.96,86.64) | 27.72  (8.97,85.71) | 77.28 | 4.79  (0.40) | 27.72  (8.91) |
| Thirst | 4 | 20.23  (7.57,54.08) | 20.09  (7.57,53.36) | 72.59 | 4.33  (0.76) | 20.09  (7.52) |
| Blood creatine phosphokinase increased | 6 | 19.91  (8.91,44.50) | 19.71  (8.89,43.71) | 106.64 | 4.30  (1.33) | 19.71  (8.82) |
| Rhabdomyolysis | 5 | 16.71  (6.93,40.31) | 16.58  (6.93,39.69) | 73.23 | 4.05  (1.02) | 16.58  (6.87) |
| Liver function test abnormal | 4 | 15.32  (5.73,40.97) | 15.23  (5.73,40.43) | 53.18 | 3.93  (0.69) | 15.22  (5.69) |
| Dyspepsia | 18 | 12.75  (7.98,20.39) | 12.39  (7.86,19.53) | 188.94 | 3.63  (2.28) | 12.39  (7.75) |
| Pancreatitis | 4 | 10.89  (4.07,29.12) | 10.83  (4.08,28.75) | 35.69 | 3.44  (0.57) | 10.83  (4.05) |
| Abdominal discomfort | 14 | 10.08  (5.93,17.13) | 9.86  (5.88,16.55) | 111.79 | 3.30  (1.88) | 9.86  (5.80) |
| Aspartate aminotransferase increased | 5 | 9.43  (3.91,22.75) | 9.36  (3.91,22.41) | 37.38 | 3.23  (0.78) | 9.36  (3.88) |
| Constipation | 18 | 9.04  (5.65,14.46) | 8.79  (5.58,13.86) | 124.77 | 3.14  (1.97) | 8.79  (5.50) |
| Renal impairment | 5 | 7.90  (3.28,19.06) | 7.84  (3.28,18.77) | 29.89 | 2.97  (0.69) | 7.84  (3.25) |
| Alanine aminotransferase increased | 5 | 7.75  (3.21,18.69) | 7.69  (3.21,18.41) | 29.13 | 2.94  (0.68) | 7.69  (3.19) |
| Hypoglycemia | 5 | 7.72  (3.20,18.63) | 7.67  (3.20,18.35) | 29.02 | 2.94  (0.68) | 7.67  (3.18) |
| Myalgia | 27 | 4.95  (3.36,7.28) | 4.77  (3.30,6.89) | 81.13 | 2.25  (1.51) | 4.77  (3.24) |

Note1: Ranked by ROR

Note2: Signals are detected when all the following criteria are meta ≥ 3, PRR ≥2 and Chi-Square ≥ 4, lower limit of 95% CI of ROR > 1, IC025 > 0, EBGM05 > 2.

**Supplementary Table 7** **Signal strength of adverse events at the Preferred Term (PT) level ranked by ROR**

| **Preferred Term (PT)** | **Case reports** | **ROR (95% CI)** | **PRR (95% CI)** | **Chi Square** | **IC (IC025)** | **EBGM (EBGM05)** |
| --- | --- | --- | --- | --- | --- | --- |
| High density lipoprotein decreased | 59 | 268.43  (207.07,347.97) | 264.51  (204.81,341.61) | 15191.6 | 8.02  (5.23) | 259.45  (200.14) |
| Creatinine renal clearance increased | 10 | 205.55  (110.00,384.12) | 205.04  (109.89,382.58) | 2000.14 | 7.66  (2.51) | 201.99  (108.09) |
| Blood triglycerides abnormal | 8 | 140.48  (69.95,282.12) | 140.21  (69.91,281.17) | 1094.36 | 7.12  (2.12) | 138.78  (69.10) |
| Low density lipoprotein abnormal | 4 | 111.55  (41.68,298.57) | 111.44  (41.68,297.99) | 434.22 | 6.79  (0.97) | 110.54  (41.30) |
| Blood triglycerides increased | 120 | 104.64  (87.20,125.57) | 101.55  (85.09,121.20) | 11861.8 | 6.66  (5.52) | 100.80  (84.00) |
| Low density lipoprotein increased | 34 | 70.72  (50.41,99.20) | 70.13  (50.14,98.09) | 2305.20 | 6.12  (4.07) | 69.77  (49.74) |
| Blood creatine increased | 19 | 65.68  (41.81,103.20) | 65.38  (41.70,102.50) | 1198.73 | 6.02  (3.30) | 65.07  (41.41) |
| Glomerular filtration rate decreased | 28 | 38.85  (26.78,56.37) | 38.59  (26.66,55.84) | 1022.42 | 5.27  (3.53) | 38.48  (26.52) |
| Blood creatinine increased | 125 | 29.60  (24.77,35.38) | 28.71  (24.16,34.13) | 3340.24 | 4.84  (4.29) | 28.66  (23.98) |
| Blood creatine phosphokinase increased | 43 | 21.68  (16.05,29.29) | 21.46  (15.94,28.90) | 837.94 | 4.42  (3.43) | 21.43  (15.86) |
| Myalgia | 199 | 18.68  (16.20,21.54) | 17.81  (15.55,20.39) | 3161.20 | 4.15  (3.83) | 17.78  (15.42) |
| Muscle fatigue | 5 | 18.56  (7.72,44.65) | 18.54  (7.72,44.55) | 82.86 | 4.21  (1.06) | 18.52  (7.70) |
| Myopathy | 10 | 17.58  (9.45,32.70) | 17.53  (9.44,32.58) | 155.74 | 4.13  (1.94) | 17.51  (9.41) |
| Renal function test abnormal | 5 | 17.19  (7.14,41.33) | 17.16  (7.14,41.24) | 76.03 | 4.10  (1.04) | 17.14  (7.13) |
| Liver function test abnormal | 32 | 16.03  (11.32,22.71) | 15.91  (11.27,22.48) | 446.97 | 3.99  (2.95) | 15.90  (11.22) |
| Hepatic enzyme increased | 66 | 16.00  (12.54,20.41) | 15.75  (12.40,20.02) | 911.87 | 3.98  (3.33) | 15.74  (12.34) |
| Rhabdomyolysis | 40 | 14.91  (10.92,20.36) | 14.77  (10.85,20.11) | 513.24 | 3.88  (3.01) | 14.75  (10.80) |
| Biliary colic | 3 | 13.43  (4.33,41.69) | 13.42  (4.33,41.62) | 34.46 | 3.75  (0.26) | 13.41  (4.32) |
| Protein urine present | 4 | 12.61  (4.73,33.63) | 12.60  (4.73,33.57) | 42.68 | 3.65  (0.63) | 12.59  (4.72) |
| Prothrombin time prolonged | 5 | 11.66  (4.85,28.04) | 11.65  (4.85,27.98) | 48.64 | 3.54  (0.89) | 11.64  (4.84) |
| Pancreatitis | 39 | 11.34  (8.27,15.54) | 11.23  (8.22,15.36) | 363.64 | 3.49  (2.70) | 11.23  (8.19) |
| Myositis | 6 | 10.92  (4.90,24.32) | 10.90  (4.90,24.26) | 53.93 | 3.45  (1.08) | 10.89  (4.89) |
| Tenderness | 7 | 10.09  (4.81,21.18) | 10.07  (4.80,21.12) | 57.18 | 3.33  (1.22) | 10.07  (4.79) |
| Blood cholesterol abnormal | 3 | 9.88  (3.18,30.66) | 9.87  (3.18,30.61) | 23.91 | 3.30  (0.17) | 9.87  (3.18) |
| Chromaturia | 15 | 9.76  (5.87,16.20) | 9.72  (5.87,16.12) | 117.35 | 3.28  (1.93) | 9.72  (5.85) |
| Photosensitivity reaction | 10 | 9.22  (4.95,17.15) | 9.20  (4.95,17.08) | 73.02 | 3.20  (1.53) | 9.19  (4.94) |
| Pancreatitis acute | 13 | 9.15  (5.31,15.78) | 9.13  (5.30,15.71) | 94.03 | 3.19  (1.76) | 9.12  (5.29) |
| Muscle disorder | 5 | 9.09  (3.78,21.87) | 9.08  (3.78,21.82) | 35.95 | 3.18  (0.77) | 9.08  (3.78) |
| International normalised ratio increased | 17 | 8.37  (5.20,13.48) | 8.34  (5.19,13.40) | 109.75 | 3.06  (1.88) | 8.33  (5.17) |
| Flank pain | 5 | 8.19  (3.41,19.70) | 8.18  (3.41,19.65) | 31.51 | 3.03  (0.72) | 8.18  (3.40) |
| Hypertriglyceridaemia | 3 | 7.91  (2.55,24.55) | 7.91  (2.55,24.52) | 18.09 | 2.98  (0.09) | 7.90  (2.55) |
| Blood urea increased | 8 | 6.90  (3.45,13.81) | 6.89  (3.45,13.76) | 40.25 | 2.78  (1.09) | 6.88  (3.44) |
| Cholecystitis | 5 | 6.55  (2.73,15.76) | 6.55  (2.73,15.72) | 23.49 | 2.71  (0.59) | 6.54  (2.72) |
| Hepatitis | 10 | 6.07  (3.26,11.29) | 6.06  (3.26,11.25) | 42.22 | 2.60  (1.18) | 6.05  (3.25) |
| Gout | 7 | 5.98  (2.85,12.56) | 5.97  (2.85,12.52) | 28.97 | 2.58  (0.86) | 5.97  (2.84) |
| Product residue present | 5 | 5.86  (2.44,14.08) | 5.85  (2.44,14.05) | 20.10 | 2.55  (0.51) | 5.85  (2.43) |
| Blood cholesterol increased | 17 | 5.69  (3.53,9.16) | 5.67  (3.53,9.11) | 65.34 | 2.50  (1.49) | 5.66  (3.52) |
| Renal impairment | 29 | 5.45  (3.78,7.86) | 5.42  (3.77,7.79) | 104.63 | 2.44  (1.71) | 5.42  (3.76) |
| Muscle spasms | 61 | 5.05  (3.92,6.51) | 4.99  (3.89,6.40) | 195.15 | 2.32  (1.86) | 4.99  (3.87) |
| Faeces discoloured | 7 | 4.87  (2.32,10.23) | 4.86  (2.32,10.20) | 21.49 | 2.28  (0.69) | 4.86  (2.32) |
| Aspartate aminotransferase increased | 17 | 4.83  (3.00,7.77) | 4.81  (2.99,7.73) | 51.33 | 2.27  (1.31) | 4.81  (2.99) |
| Dyspepsia | 30 | 4.80  (3.35,6.87) | 4.77  (3.34,6.82) | 89.53 | 2.25  (1.57) | 4.77  (3.33) |
| Alanine aminotransferase increased | 19 | 4.68  (2.98,7.34) | 4.66  (2.97,7.30) | 54.62 | 2.22  (1.33) | 4.66  (2.97) |
| Rash | 132 | 4.64  (3.90,5.52) | 4.52  (3.82,5.35) | 364.71 | 2.18  (1.88) | 4.52  (3.80) |
| Cholelithiasis | 10 | 4.64  (2.49,8.63) | 4.63  (2.49,8.60) | 28.49 | 2.21  (0.93) | 4.63  (2.49) |
| Rash papular | 7 | 4.63  (2.20,9.72) | 4.62  (2.20,9.69) | 19.87 | 2.21  (0.65) | 4.62  (2.20) |
| Muscular weakness | 33 | 4.45  (3.16,6.27) | 4.42  (3.15,6.21) | 87.55 | 2.14  (1.51) | 4.42  (3.14) |
| Pollakiuria | 12 | 4.42  (2.51,7.79) | 4.41  (2.51,7.76) | 31.64 | 2.14  (1.00) | 4.41  (2.50) |
| Jaundice | 8 | 4.38  (2.19,8.77) | 4.38  (2.19,8.74) | 20.83 | 2.13  (0.71) | 4.37  (2.19) |
| Pain in extremity | 67 | 3.44  (2.70,4.38) | 3.40  (2.68,4.31) | 114.12 | 1.77  (1.36) | 3.40  (2.67) |

Note1: Ranked by ROR

Note2: Signals are detected when all the following criteria are meta ≥ 3, PRR ≥2 and Chi-Square ≥ 4, lower limit of 95% CI of ROR > 1, IC025 > 0, EBGM05 > 2.
